# Supplementary figures and images for: Periodic propagating waves coordinate RhoGTPase network dynamics at the leading and trailing edges during cell migration
Source: eLife. 2020 Jul 24;9:e58165. doi: 10.7554/eLife.58165 (PMC7380942; doi:10.7554/eLife.58165)

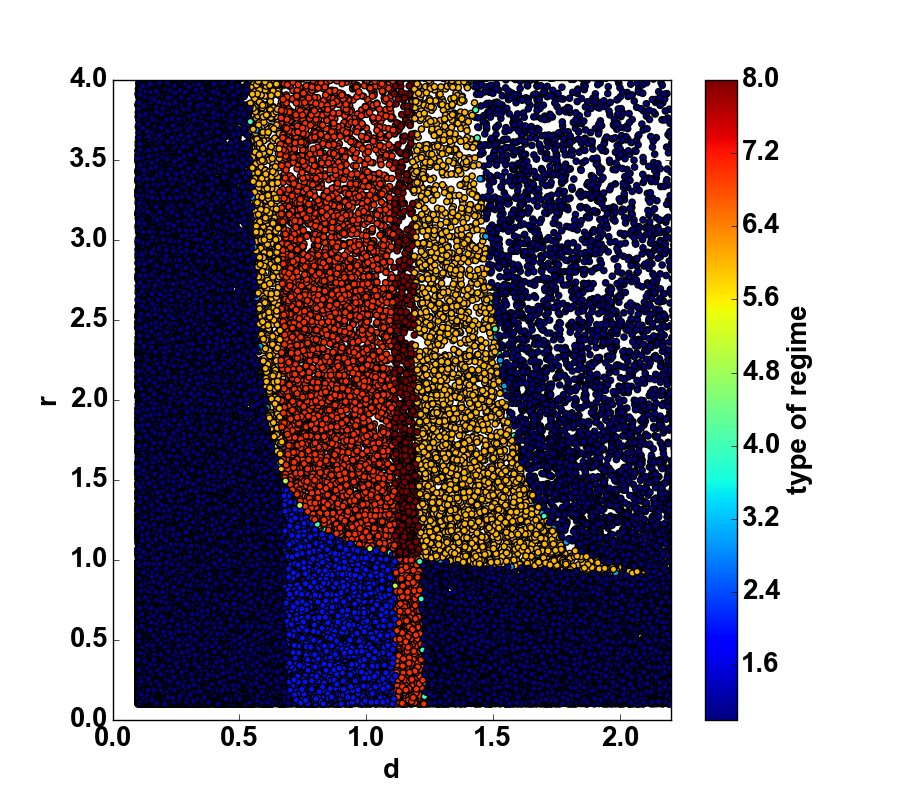

Supplement: Source code 1. — See ‘description.txt’ file in the zip-archive for details. [file elife-58165-code1.zip › bifurcation_diargams/2D_bifurcation_diagrams/2D_scan.png]
