# Supplementary material for: Periodic propagating waves coordinate RhoGTPase network dynamics at the leading and trailing edges during cell migration
Source: eLife. 2020 Jul 24;9:e58165. doi: 10.7554/eLife.58165 (PMC7380942; doi:10.7554/eLife.58165)
Supplement: Supplementary file 1. [file elife-58165-supp1.docx]

| **Supplementary file 1.** **Quantitative mass spectrometry data by (Byrne, K. M. et al, 2016) used to populate protein abundances in our mathematical model.** | | | |
| --- | --- | --- | --- |
| **Gene name** | **Protein name** | **Copy number intensity** | **Concentration (nM)*** |
| PAK2 | Serine/threonine-protein kinase PAK 2 | 661552 | 27.00 |
| RAC2 | Ras-related C3 botulinum toxin substrate 2 | 277301 | 11.09 |
| RAC1 | Ras-related C3 botulinum toxin substrate 1; Ras-related C3 botulinum toxin substrate 3 | 3984262 | 160.00 |
| RHOT1 | Mitochondrial Rho GTPase 1;Mitochondrial Rho GTPase | 7391 | 0.30 |
| RHOC | Rho-related GTP-binding protein RhoC | 530247 | 21.21 |
| RHOA | Transforming protein RhoA;Rho-related GTP-binding protein RhoC | 4013548 | 161.00 |
| RHOG | Rho-related GTP-binding protein RhoG | 490041 | 19.60 |
| RHOT2 | Mitochondrial Rho GTPase 2 | 7850 | 0.31 |
| RHOF | Rho-related GTP-binding protein RhoF | 25597 | 1.02 |
|  |  |  |  |
|  |  |  |  |
| ROCK1 | Rho-associated protein kinase 1 | 13858 | 0.55 |
| ROCK2 | Rho-associated protein kinase 2 | 62134 | 2.49 |
| DIAPH1 | Protein diaphanous homolog 1;Diaphanous homolog 1 (Drosophila), isoform CRA_a | 189210 | 7.57 |
| DIAPH2 | Protein diaphanous homolog 2;Diaphanous homolog 2 (Drosophila), isoform CRA_c;Diaphanous homolog 2 (Drosophila), isoform CRA_a | 8292 | 0.33 |
| DIAPH3 | Protein diaphanous homolog 3 | 19067 | 0.76 |

^*^ Concentrations were calculated assuming an average cell volume $4\cdot{10}^{-14}$ L.

**References**

Byrne, K. M. *et al.* Bistability in the Rac1, PAK, and RhoA Signaling Network Drives Actin Cytoskeleton Dynamics and Cell Motility Switches. *Cell Syst* **2**, 38-48, doi:10.1016/j.cels.2016.01.003 (2016).
