# Supplementary material for: Periodic propagating waves coordinate RhoGTPase network dynamics at the leading and trailing edges during cell migration
Source: eLife. 2020 Jul 24;9:e58165. doi: 10.7554/eLife.58165 (PMC7380942; doi:10.7554/eLife.58165)
Supplement: Supplementary file 2. [file elife-58165-supp2.docx]

| **Supplementary file 2.** **Parameter values (Eq. 12).** | | | | |
| --- | --- | --- | --- | --- |
| **Parameter** | **Value** |  | **Parameter** | **Value** |
| $\gamma_{Rho}^{DIA}$ | 5.0526 |  | $k_{a}^{DIA}$ | 2 |
| $\gamma_{Rho}^{ROCK}$ | 22.7795 |  | $k_{a}^{ROCK}$ | 2 |
| $v_{a}^{DIA}$ | 19.2456 1/s |  | $v_{a}^{PAK}$ | 0.0186 1/s |
| $v_{i}^{DIA}$ | 10.5262 1/s |  | $k_{a}^{PAK}$ | 0.288 |
| $k_{i}^{DIA}$ | 0.0158 |  | $v_{i}^{PAK}$ | 0.089 1/s |
| $v_{a}^{ROCK}$ | 0.2470 1/s |  | $k_{i}^{PAK}$ | 0.16 |
| $v_{i}^{ROCK}$ | 9.5818 1/s |  | $\gamma_{DIA}^{Rho}$ | 100 |
| $k_{i}^{ROCK}$ | 0.0395 |  | $k_{DIA}^{Rho}$ | 3 |
| $v_{GEF}^{Rho}$ | 0.4902 1/s |  | $\gamma_{DIA}^{Rac}$ | 7.8 |
| $k_{GEF}^{Rho}$ | 0.3591 |  | $k_{DIA}^{Rac}$ | 0.055 |
| $v_{GAP}^{Rho}$ | 0.7707 1/s |  | $\gamma_{ROCK}^{Rac}$ | 10 |
| $k_{GAP}^{Rho}$ | 0.0218 |  | $k_{ROCK}^{Rac}$ | 0.05 |
| $v_{GEF}^{Rac}$ | 0.1118 1/s |  | $k_{Rho}^{DIA}$ | 0.04 |
| $k_{GEF}^{Rac}$ | 0.0275 |  | $k_{Rho}^{ROCK}$ | 1.3 |
| $v_{GAP}^{Rac}$ | 0.5495 1/s |  | $\gamma_{PAK}^{Rho}$ | 0.025 |
| $k_{GAP}^{Rac}$ | 0.0109 |  | $k_{PAK}^{Rho}$ | 0.012 |
| $\gamma_{Rac}^{PAK}$ | 6.7 |  | $k_{Rac}^{PAK}$ | 0.65 |
| $\gamma_{PAK}^{Rac}$ | 1 |  | $k_{PAK}^{Rac}$ | 0.1 |
| $d_{Rac}$ | 0.0005 1/s |  | $d_{Rho}$ | 0.0005 1/s |
| $d_{RacD}$ | 0.0005 1/s |  | $d_{RhoD}$ | 0.0005 1/s |
| $d_{h}$ | 1.1 |  | $d_{l}$ | 0.8 |
| $r_{h}$ | 1.85 |  | $r_{l}$ | 0.5 |
| $X_{l}$ | 0.8 |  | $p$ | 1 |
| $\left[ ATP \right]/K_{d}^{ATP}$ | 112 |  | $K_{I}^{ROCK}$ | 220 nM |
